# Inhibition of BMP signaling in P-Cadherin positive hair progenitor cells leads to trichofolliculoma-like hair follicle neoplasias

**DOI:** 10.1186/1423-0127-18-92

**Published:** 2011-12-14

**Authors:** Lixin Kan, Yijie Liu, Tammy L McGuire, Michael A Bonaguidi, John A Kessler

**Affiliations:** 1 Department of Neurology, Northwestern University Feinberg School of Medicine, Chicago IL 60611, USA; 2 Institute for Cell Engineering, Johns Hopkins University School of Medicine, Baltimore, Maryland 21205, USA; 3 Department of Neurology, Johns Hopkins University School of Medicine, Baltimore, Maryland 21205, USA

**Keywords:** Transgenic Mice, Nse-Noggin, bone morphogenetic protein (BMP), trichofolliculoma

## Abstract

**Background:**

Skin stem cells contribute to all three major lineages of epidermal appendages, i.e., the epidermis, the hair follicle, and the sebaceous gland. In hair follicles, highly proliferative committed progenitor cells, called matrix cells, are located at the base of the follicle in the hair bulb. The differentiation of these early progenitor cells leads to specification of a central hair shaft surrounded by an inner root sheath (IRS) and a companion layer. Multiple signaling molecules, including bone morphogenetic proteins (BMPs), have been implicated in this process.

**Methods:**

To further probe the contribution of BMP signaling to hair follicle development and maintenance we employed a transgenic mouse that expresses the BMP inhibitor, Noggin, to disrupt BMP signaling specifically in subset of hair follicle progenitors under the control of neuron specific enolase (Nse) promoter. We then studied the skin tumor phenotypes of the transgenic mice through histology, immunohistochemistry and Western Blotting to delineate the underlying mechanisms. Double transgenic mice expressing BMP as well as noggin under control of the Nse promoter were used to rescue the skin tumor phenotypes.

**Results:**

We found that the transgene is expressed specifically in a subpopulation of P-cadherin positive progenitor cells in Nse-Noggin mice. Blocking BMP signaling in this cell population led to benign hair follicle-derived neoplasias resembling human trichofolliculomas, associated with down-regulation of E-cadherin expression and dynamic regulation of CD44.

**Conclusions:**

These observations further define a critical role for BMP signaling in maintaining the homeostasis of hair follicles, and suggest that dysregulation of BMP signaling in hair follicle progenitors may contribute to human trichofolliculoma.

## Background

The skin is a barrier that protects against the physical, chemical, and thermal assaults of the environment. To serve these functions, epidermis generates an elaborate array of supportive appendages, including hair follicles (HFs), sebaceous glands, sweat glands, and nails [1]. Many conserved signal molecules, such as WNT[2], NOTCH[3], FGF[4], Hedgehog[5] and BMP[6], are involved in orchestrating the development and maintenance of this important organ. Not surprisingly, disruption of BMP signaling has been implicated in an array of skin disorders.

BMP signaling plays essential roles in many biological processes in numerous types of cells and tissues during embryonic development and adult life [7]. BMP actions are regulated *in vivo *in a time and location-dependent manner by proteins such as noggin, gremlin, chordin and others that antagonize BMP signaling by directly binding BMPs and their immediate downstream mediators, thus blocking ligand activity [8]. Not surprisingly, the phenotypes generated from disrupting BMP signaling through loss-of-function or gain-of-function mutations are temporally, spatially, and dosage-dependent. For example, germline mutation of BMP2 or BMP4 leads to embryonic lethality [9,10], whereas inhibition of BMP signaling by overexpressing Noggin under control of different promoters, or by conditional knockout of BMP receptor subunits, leads to variable phenotypes. Many of these mutant animals show strong cutaneous phenotypes that resemble human skin disorders. Further study along this line will deepen our understanding of the normal role of BMP signaling in skin and supportive appendages.

Here we utilized transgenic mice that overexpress noggin under control of the neuron-specific enolase (Nse) promoter [11] to further probe the role of BMP signaling in orchestrating proliferation and differentiation of epidermal progenitors. We found that overexpression of Noggin in P-cadherin positive hair progenitor cells led to benign hair follicle-derived neoplasias resembling human trichofolliculomas.

Trichofolliculoma (also called folliculoma, or hair follicle nevus) is a benign highly structured hamartoma of the pilosebaceous unit. The morphologic features of trichofolliculoma are variable, reminiscent of the anagen, catagen, and telogen phases of a normal hair follicle in its cycle. However, the follicular epithelium usually exhibits a distinct granular cell layer similar to the normal follicular infundibulum. Follicles or follicle-like structures branch to form secondary or tertiary units. Trichokeratin, not real hair, may be found enclosed in the follicle matrix cells, and the stroma is moderately cellular and loosely organized similar to that found in the normal follicle. These characteristic histological features distinguish this disorder from similar skin disorders such as dilated pore of winer, trichoepithelioma, folliculosebaceous cystic hamartoma [12], pilomatricomas or sebaceous trichofolliculoma [13]. However, the precise etiology of trichofolliculoma is still unknown.

Our current study of a trichofolliculoma-like phenotype in Nse-Noggin mice indicated that blocking BMP signaling in hair follicle progenitors is associated with down-regulation of E-cadherin expression and subsequent dosage dependent up-regulation of CD44. Complimentary to a previous report [14], we also observed dysregulation of β-catenin signaling in tumor cells. This study not only provides additional evidence of the importance of BMP signaling in maintaining the homeostasis of hair follicles, but also may help to further understand the pathophysiology of human skin disorders, especially trichofolliculomas.

## Materials and Methods

### Animal study procedures

Previously generated Nse-Noggin and Nse-BMP4 mice were used in this study [15,16]. All animal experiments in this study were approved by the Animal Care and Use Committee at Northwestern University.

### Histology and immunohistochemistry

Hematoxylin and eosin staining was performed on fixed tissue sections using Harris Modified Hematoxylin and Eosin Y Solution (Sigma, St. Louis, MO), according to the manufacturer's instructions. Immunostaining was done using standard protocols [17]. Briefly, sections were fixed with 4% paraformaldehyde in PBS. Non-specific binding was blocked with 10% normal serum diluted in 1% bovine serum albumin (BSA, Jackson Lab, USA) and 0.25% Triton X-100 for one hour in room temperature. The sections were then incubated with primary antibodies diluted with 1% BSA + 0.25% Triton X-100 at 4°C overnight. The sections were then incubated with appropriate secondary antibodies (Cy3 or Cy2 conjugated antibodies (Jackson Lab) diluted with 1% BSA + 0.25% Triton X-100 or Alexa Fluor 488, Alexa Fluor 594, and Alexa 647 (1:1000, Invitrogen)) in the dark at room temperature for 2 hours. Counterstaining was then performed with DAPI (1:5000). Fluorescent images were processed by Adobe Photoshop. Anti-Ki67 (Novus Biologicals), anti-total β-cat (Santa Cruz Biotechnology Inc), anti-phospho β-cat (Thr41/Ser45) (cell signaling), anti-active β-cat (clone 8E7, Millipore), and NSE (BioGenex), anti-K14 (covance) are used in this study.

### Western blot analysis

Protein levels of CD44 and P-cad in tissue were measured by Western blotting. Tissues were homogenized in protein extract buffer (Roche) and homogenized samples (20 μg of protein) were subjected to 4-20% SDSPAGE gradient gel (Bio-Rad) under reducing conditions. The CD44 was identified by Rat anti-mouse CD44 (BD Bioscience) and P-cad was identified by mouse anti-p-cad antibody (BD Bioscience). The membranes were incubated with the secondary antibodies (Biotinylated goat anti-rat IgG and Biotinylated goat anti-mouse IgG1) (Santa Cruz Biotechnology Inc) for 1 hr at room temperature. Blots were developed by the ECL Western blotting detection reagents (Perkin-Elmer).

### Statistical Analyses

Values are expressed as means ± standard deviation of the mean, and p < 0.05 is considered to be statistical significance. Intergroup comparisons were made using two-way ANOVA. The photographs shown represent the results obtained from three independent experiments.

## Results

### The noggin transgene is expressed in P-cadherin positive hair progenitor cells in Nse-Noggin mice

Noggin overexpression under different promoters leads to a spectrum of phenotypes due to differing temporal and spatial patterns of transgene expression [18-21]. We initially constructed transgenic animals that overexpress the BMP inhibitor noggin under control of the Nse promoter (Nse-noggin) to examine the role of BMP signaling in development of the brain. We noticed that Nse-Noggin mice have a dramatic hair phenotype [16] and that substantial numbers of adult Nse-noggin mice also developed skin tumors.

**Figure 1:**
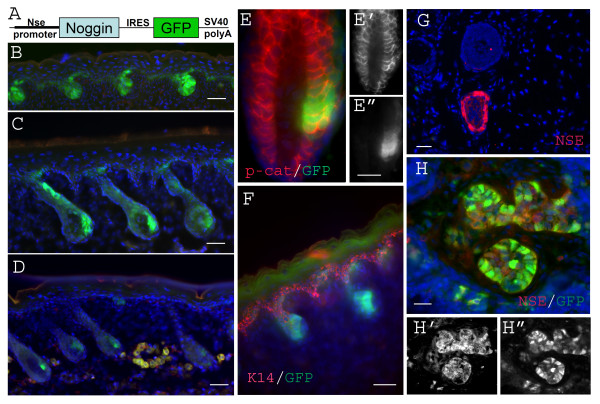
**The Noggin transgene is expressed in P-cadherin positive hair progenitor cells in Nse-Noggin mice from late embryonic stages onward**. (A) Schematic structure of Nse-Noggin transgene. (B-D) typical images of tail skin of Nse-Noggin mice show the specific transgene (GFP) expression pattern. GFP positive cells are detected in hair buds from E16 (B) In postnatal animals (P0(C) & P6(D)) transgene expression is also observed only in subpopulations of hair follicles (C&D). (E-F) double staining confirms that the transgene is expressed in hair progenitors (P-cadherin Positive, E-E") but not in fully differentiated epithelium (K14 positive, F). (E) shows typical GFP/P-cadherin double staining of a hair follicle from an adult Nse-Noggin animal. E'& E" are the split channels of P-cadherin and GFP, respectively. (F) shows typical GFP/K14 double staining of a hair follicle from an E18 Nse-Noggin mouse. (G) shows typical NSE staining of a hair follicle from an adult WT animal. Note that NSE is highly expressed in a subpopulation of hair follicle cells. (H-H") shows typical GFP/NSE double staining of well developed tumor section. H'& H" are the split channels of NSE and GFP, respectively. Bar = 40 μm.

To begin to understand how inhibition of BMP signaling in this mouse model leads to tumorigenesis, we first examined the pattern of transgene expression in skin. Since the transgene has an IRES-GFP tag in this line, we could easily track transgene expression cells by epifluorescence of GFP (Figure. 1A). Previous studies indicated that the transgene is over-expressed under this promoter in neurons from the late embryonic stage onward [15]. Our current study found that transgene was also expressed in a subpopulation of hair follicle cells from late embryonic stages onward (Figure 1B-D). Double staining for GFP and for either keratin 14 (K14, a fully differentiated keratinocyte marker) or P-cadherin, a hair follicle progenitor marker in skin [22], indicated that the transgene is expressed by a subpopulation of hair follicle progenitor cells, but not by fully differentiated keratinocytes (Figure 1E&F). To clarify whether this transgene expression pattern reflects the endogenous Nse promoter activity, we stained WT skin samples with a specific monoclonal antibody that recognizes endogenous NSE protein. We found that endogenous NSE is highly expressed in a subpopulation of hair follicle cells (Figure 1G). Consistently, extensive co-localization of NSE and the GFP transgene tag was observed in tumor cells (Figure 1H).

### Blocking BMP signaling in hair progenitor cells induces benign hair follicle-derived neoplasias resembling trichofolliculomas

**Figure 2:**
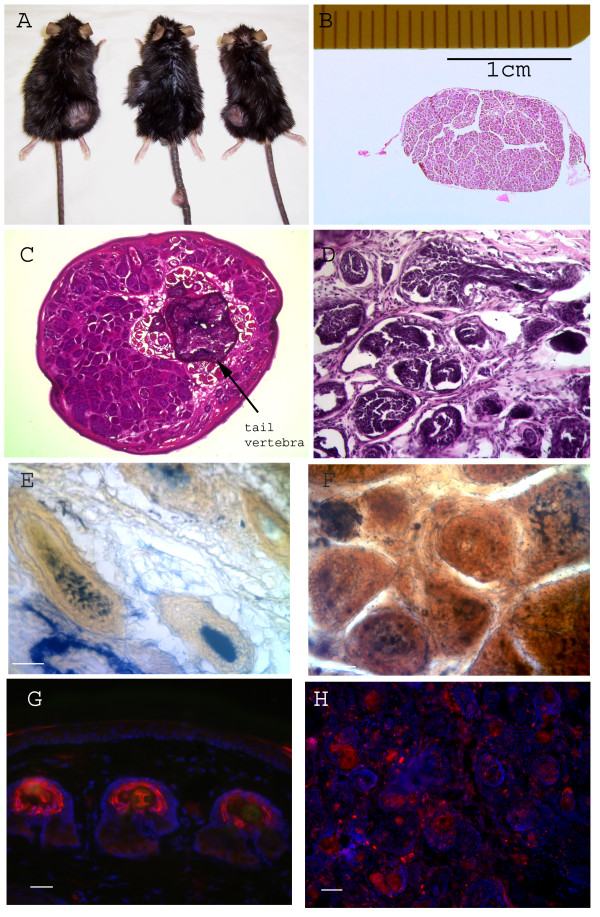
**Blocking BMP signaling in hair progenitor cells induces benign hair follicle-derived neoplasias resembling trichofolliculomas**. (A) Gross image show the sizes and locations of neoplasias in three different mice. Note that the sizes and locations vary in different mice. Black arrows point to neoplasias. (B-C) typical low power H&E images from a fully developed neoplasia from back skin (B) and tail (C). Note the well-formed fibrous sheath that surrounds the outer surface of tumor. Also note that a fully developed neoplasia from back skin was over 1 cm in diameter. Neoplasias that grow on the tail are relatively small, and are not well wrapped(C), but nevertheless both show typical histological features of trichofolliculomas. (D) High power H&E image from a tail tumor shows multiple abnormal secondary hair follicle-like structures surrounded by fibroblast-like stroma cells. (E-H) two independent alkaline phosphatase assays indicated that dermal papillae are present in tumor. (E&F) tested the enzymatic activity of alkaline phosphatase in WT tail skin (E) and tumor (F). Note the strong blue ALP staining in WT hair follicle and tumor (yellow to brown in (E&F) is background staining). (G&H) detect the alkaline phosphatase antigen in WT tail skin (G) and tumor (H) through standard IHC. Note, similar to (E&F), strong ALP staining was detected in both WT hair follicle and tumor. Bar = 40 μm in (D-H).

**Figure 3:**
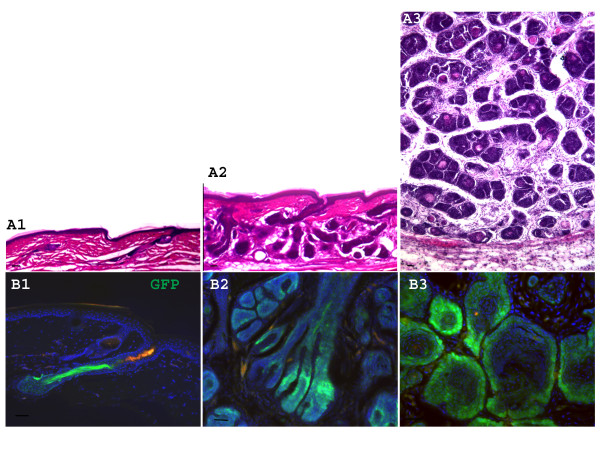
**Blocking BMP signaling in hair progenitor cells induces ordered tumorigenesis**. (A1-A3) H&E images from different time points of Nse-Noggin animals show morphological features of neoplasias at different stages of tumorigenesis. Note the progressive development of abnormal secondary hair follicles. (B1-B3) show the typical transgene expression pattern at different stages of tumorigenesis. Note that transgene expression is restricted to hair follicle cells and not other cells. Bar = 40 μm.

As the transgenic mice matured they developed skin tumors. The size and location of tumors varied greatly, and some mice developed multiple tumors. Detailed histological study found that these tumors consistently recapitulated the characteristic histological features of human trichofolliculomas, i.e., the follicular epithelium exhibits a distinct granular cell layer similar to the normal follicular infundibulum. Follicles or follicle-like structures branch to form complex secondary or tertiary units, and such secondary or tertiary follicles display features of lower segment differentiation, such as a hair bulb, or the presence of a hair shaft. The stroma, on the other hand, is loosely organized similar to that found in the normal follicle (Figure 2). Alkaline phosphatase staining confirmed that dermal papillae are present in tumor (Figure 2E-H). Detailed histological study of tumors taken from mice at different stages of tumorigenesis revealed that blocking BMP signaling in hair progenitors induced ordered transformation, hyperplasia, and finally neoplasias resembling trichofolliculomas (Figure 3). This slow and orderly tumorigenesis process may take a few months to finish.

### Nse-BMP4 rescued the tumor phenotype in Nse-Noggin mice

**Figure 4:**
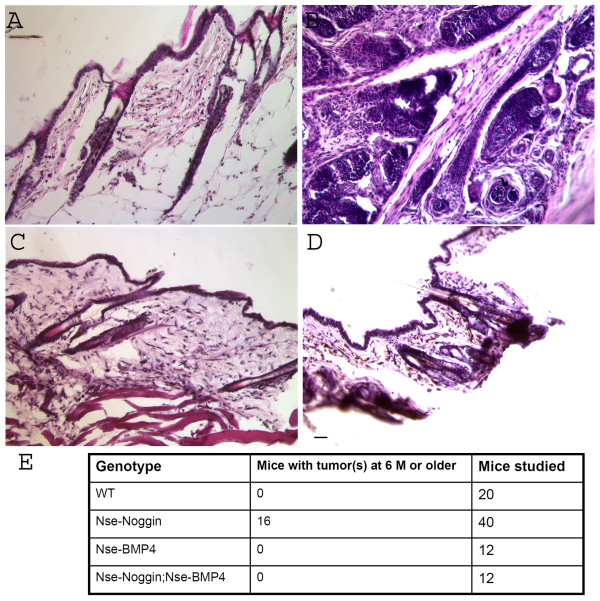
**Nse-BMP4;Nse-Noggin double transgenic mice show only subtle hyperplasia of hair follicles**. Representative H&E staining from WT (A), Nse-Noggin (B), Nse-BMP4 (C) and Nse-Noggin; Nse-BMP4 double transgenic mice (D) show histological features in different lines of animals. Note that crossing the Nse-Noggin mice with Nse-BMP4 mice rescued the tumor phenotype seen in Nse-Noggin mice (comparing B&D), but subtle hyperplasia of hair follicles is still observable in (D). Bar = 40 μm (E) summary of severity of tumorigenesis in different lines.

To further prove that the observed tumor phenotype is BMP signaling dependent, not due to the nonspecific positional effort of transgene integration, we generated Nse-BMP4;Nse-Noggin double transgenic mice by mating Nse-BMP4 with Nse-Noggin mice. The rationale of this experiment is that if the tumor phenotype is caused by specifically inhibiting BMP signaling, overexpression of BMP4 under the same promoter could at least partially block the inhibition. We found none of the Nse-BMP4;Nse-Noggin double transgenic mice develop observable tumors. Histological study confirmed this observation, even though subtle hyperplasia of hair follicles is still observable in double transgenic mice (Figure 4). This data confirmed that noggin exerted its effects specifically by inhibiting BMP signaling.

### Mechanisms of Noggin induced tumorigenesis

**Figure 5:**
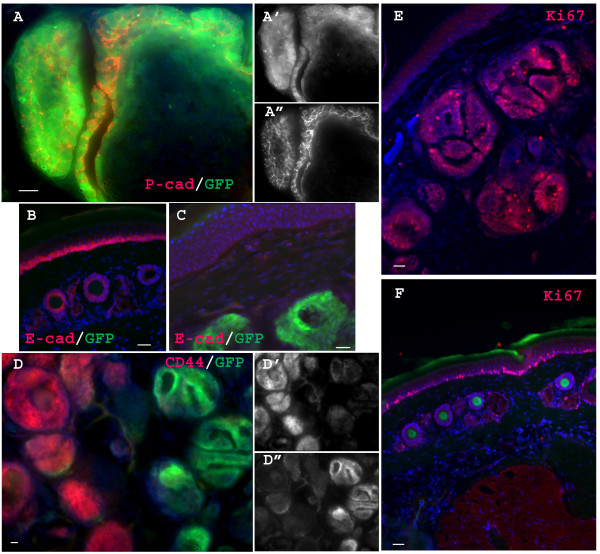
**Blocking BMP signaling in hair progenitor cells disrupts expression of key molecules that are essential for normal morphogenesis of hair follicles**. (A) shows typical GFP/P-cad double staining of a neoplasia from an Nse-Noggin mouse. Note that P-cad is over expressed in GFP positive cells. (A'& A") show the split channel of GFP and P-cad, respectively. (B&C) show typical GFP/E-cad double staining of a tail neoplasia (B) and a section of WT tail tissue (C). Note that E-cad is normally expressed in basal epithelia and inner root sheath of tail. This normal expression is dramatically reduced not only in tumor cells but also in the basal epithelia adjacent to tumor. (D) shows typical GFP/CD44 double staining of a neoplasia. Note the clear inverse relationship of GFP and CD44 expression, i.e., secondary hair follicles that have strong GFP expression always have low CD44 expression. (D'&D") show the split channels of CD44 and GFP, respectively. (E&F) show typical Ki67 staining of a tail neoplasia (E) and a section of WT tail tissue (F). Note that, normally, a subpopulation of basal epithelia has strong expression of Ki67, but very few hair follicle cells are Ki67 positive. Ki67 positive cells are dramatically increased in the tumor. In contrast, Ki67 expression is reduced in the basal epithelia that adjacent to tumor. Bar = 40 μm.

When E-cadherin is transgenically elevated in mouse skin, hair follicle morphogenesis is blocked, suggesting that E-cadherin down-regulation is a critical event in the adhesion dynamics governing morphogenesis of hair follicles [23]. To help clarify the specific molecular and cellular mechanisms underlying tumorigenesis in Nse-Noggin mice, we double stained sections of tumor for E-Cadherin, a normal basal epithelium marker, and for P-Cadherin, a marker of hair progenitor cells. Expression of P-Cadherin was significantly up-regulated in GFP positive cells. By contrast, E-Cadherin was down-regulated not only in GFP positive tumor cells, but also in normal basal epithelia adjacent to the tumor (Figure 5). Thus inhibition of BMP signaling differentially disrupts the composition of cell adhesion proteins that are essential for maintaining the architecture of normal skin. The net effect is to prevent hair progenitor cells from differentiating, while maintaining the proliferation of keratinocyte lineage. Consistently, Ki67, a cell proliferation marker, was dramatically up-regulated in tumor cells, indicating that tumor cells remained in the cell cycle and were not terminally differentiated (Figure 5).

**Figure 6:**
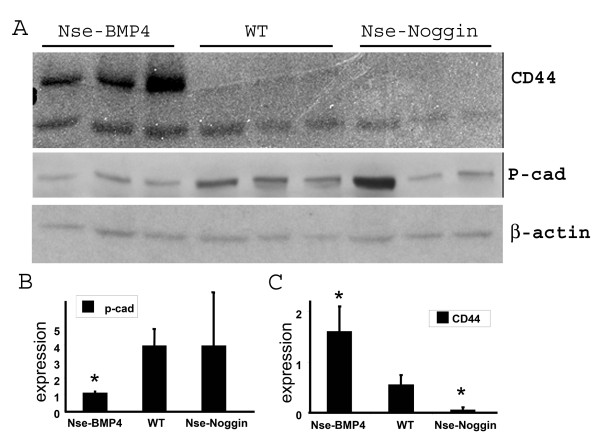
**Western blot analysis confirms that expression of CD44 and P-cad is differentially regulated**. (A)Western Blotting using skin lysates from Nse-BMP4, WT and Nse-Noggin mice. Each lane represents lysate from an individual animal. Nse-BMP4 (lane 1-3), WT (lane 4-6), Nse-Noggin (lane 7-9). Lane 7 represents lysate from a well-developed tumor, while lane 8&9 represent pre-neoplasia lysates from two individual Nse-Noggin mice. Top panel shows CD44 expression. Notice that CD44 is not up-regulated in Nse-Noggin tumors generally, which is consistent with the double-staining finding in (Figure 3D). Middle panel shows P-cad expression. Note that P-cad expression is dramatically up-regulated only in well-developed tumor. (B&C) quantify the protein expression of P-cad and CD44, respectively. *, differs from WT by ANOVA at p < 0.01.

To further explore different aspects of tumorigenesis, we double stained for GFP and CD44, a transmembrane receptor that is implicated in cell migration [24]. Previous studies have indicated that CD44 overexpression and activation plays a role in the trafficking of metastatic cancer to distant sites [24]. This receptor was also dynamically regulated by BMP signaling during tumorigenesis in a dosage-depend way, i.e., cells that expressed low levels of GFP/Noggin had strong CD44 staining while cells that expressed high levels of GFP/noggin had minimal CD44 expression. The overall CD44 expression level, however, was not up-regulated in tumor. This finding was confirmed by Western blotting (Figure 6).

**Figure 7:**
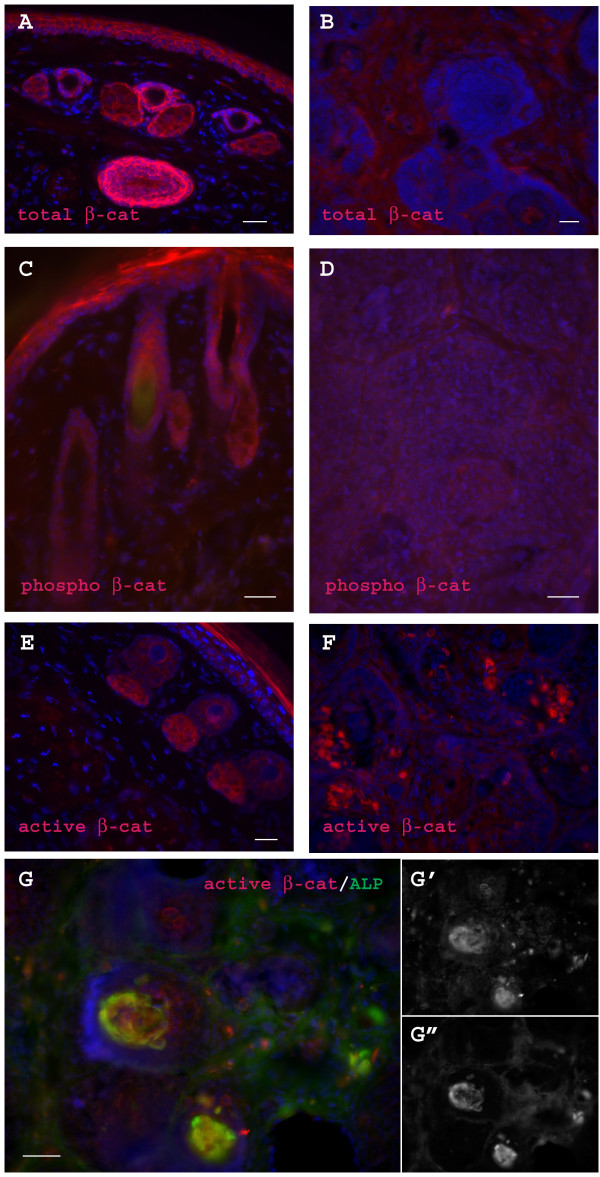
**β-catenin signaling is dysregulated in the tumor**. (A&B) show that total β-catenin is down-regulated in tumor cells. Detection of total β-catenin antigen in WT tail skin (A) and tumor (B) through standard IHC. Note that total β-catenin antigen is down-regulated in the tumor. (C&D) show that phosphorylated β-catenin is down-regulated in tumor cells. Detection of phosphorylated β-catenin antigen in WT tail skin (A) and tumor (B) through standard IHC. Note that phosphorylated β-catenin antigen is down-regulated in the tumor. (E&F) show that active β-catenin is up-regulated in a subpopulation of cells. Detection of active β-catenin antigen in WT tail skin (A) and tumor (B) through standard IHC. Note that active β-catenin antigen is up-regulated in a small population of cells, and slightly down-regulated or unchanged in the majority of tumor cells. (G) double staining found that high levels of stable β-catenin almost exclusively co-localized with a dermal papillae marker (ALP). G'& G" are the split channels of active β-catenin and ALP, respectively. Bar = 40 μm.

A previous report indicated that overexpressing stabilized β-catenin specifically in the epidermis and follicle outer root sheath induced two types of tumors that resemble trichofolliculomas and pilomatricomas[14]. To clarify if β-catenin is dysregulated in our model, we preformed additional immunostaining for total β-catenin, phosphorylated β-catenin, and active/stable β-catenin, and we found that both total β-catenin and phosphorylated β-catenin were down-regulated in the tumor, especially in the hair follicle derived cells (Figure 7A-D). In contrast, even though no obvious change was found in the majority of tumor cells, active/stable beta-catenin was dramatically up-regulated in a minor subpopulation of cells (Figure 7E&F). Further study found that the high levels of stable β-catenin almost exclusively co-localized with a dermal papillae marker (ALP) (Figure 7G).

## Discussion

Mammalian skin begins as a single sheet of multipotent ectodermal cells. The development of a hair follicle requires a series of coordinated changes in the behavior of the targeted cells within an epithelial sheet [1]. The process must be accompanied by alterations in the proliferation, polarity, shape, and adhesiveness of selected cells. BMP signaling plays a major role in numerous stages of this process. Consequently loss-of-function mutations in BMP signaling at different developmental stages or locations within the epithelium leads to strikingly different phenotypes, consistent with many prior studies that have shown developmental changes in the functions of growth factors [25].

Conditional null mutation of the BMP type IA receptor (BMPRIA) through cre-mediated recombination driven by different promoters leads to different phenotypes. For example, knockout of *BMPR-IA *through *K14-Cre *mediated recombination leads to severe defects in the inner root sheath and hair shaft differentiation, retardation of catagen and formation of hair follicles-derived tumors with age [26]. By contrast, knockout of *BMPR-IA *through *Emx1-Cre *mediated recombination, leads to defects in the inner root sheath differentiation, formation of cysts in the hair canal [6]. Finally knockout of *BMPR-IA *through *En1-Cre *mediated recombination leads to severe defects in the inner root sheath and hair shaft formation in ventral hair follicles [27].

Interestingly, both pro- and anti-tumor effects of BMP signaling have been reported in the literature. For example, loss-of-function mutations in human of either *MADH4 *(which encodes SMAD4, the downstream transcription factor that mediates the BMP signaling) or BMPRIA are associated with juvenile polyposis syndrome [28]. Further, conditional knockout of Smad4 through *MMTV-Cre *mediated recombination leads to squamous cell carcinoma formation [29]. BMP signaling also exerts anti-tumor effects in *cytokeratin IV-BMP-4 *transgenic mice skin that resist the TPA (phorbol ester 12-O-tetradecanoylphorbol-13-acetate) induced papillomas and squamous cell carcinomas formation [30]. By contrast, BMP proteins are highly over-expressed in human non-small cell lung carcinoma and recombinant BMP-2 enhances the growth of tumors formed from A549 cells injected subcutaneously into nude mice [31]. Further, BMP-4 mRNA is preferentially overexpressed in poorly differentiated gastric cancer [32], and overexpression of BMP-2/4, -5 or BMPR-IA are all associated with malignancy of oral epithelium and may be involved in the metastasis of oral carcinoma cells [33]. Thus, the biological responses to BMP signaling are cell type and/or context dependent.

More importantly, even overexpressing Noggin in skin under different promoters leads to a spectrum of phenotypes. For example, *Msx2-Noggin *transgenic mice lack external hairs due to severe alterations in hair shaft formation [34]; *K5-Noggin *transgenic mice have retarded epidermal differentiation and reduced apoptosis [19]; *K14-Noggin *transgenic mice have a thickened epidermis with formation of compound vibrissa hair follicles [35]. Note that none of these studies reported tumorigenesis in these mice.

This study using the Nse-Noggin mouse model, as well as a prior study involving noggin overexpression in the skin [36], provides evidence that inhibition of BMP signaling may play a role in tumorigenesis. Specifically, we found that inhibition of BMP signaling by overexpression of Noggin in hair follicles resulted in formation of tumors that resemble trichofolliculomas, a relatively uncommon hair follicle-derived neoplasia.

Presumably the phenotype of Nse-Noggin mice is different from the other cutaneous Noggin transgenic mice because of differences in the expression patterns of the transgenes. In fact, in both *K14-Noggin *and *K5*-*Noggin *transgenic mice the transgene is overexpressed predominately in fully differentiated keratinocytes instead of hair follicle progenitors as in the Nse-Noggin mice. Similarly, in *Msx2-Noggin *transgene mice expression of transgene is also largely seen in differentiated epithelial cells of the precortex and hair shaft region. Our observations that hair progenitor cells overexpressing transgene remain in the cell cycle and down regulate E-cadherin expression (both are key events for tumorigenesis) further argue that this specific transgene pattern might be crucial for the tumorigenesis. Importantly, mating these mice with BMP4 transgenic mice under the same promoter rescued the tumor phenotype indicating that inhibition of BMP signaling mediated the effects of the noggin transgene.

More interestingly, significantly different phenotypes are even found in transgenic mice that used the same promoter. For example, both Plikus M et al. [35] and Sharov AA et al. [36] studied the phenotypes of K14-Noggin transgenic mice. Sharov AA et al. found that transgenic mice (on an FVB background) developed spontaneous hair follicle-derived tumors which resemble human trichofolliculomas. In contrast, Plikus et al. observed thickened skin epidermis, increased hair density, altered hair types, faster anagen re-entry, and formation of compound vibrissa follicles in their transgenic mice (C57BL/6J background). Mouse strain differences could have contributed to altered tumorigenicity of K14-Noggin, however, we observed similar tumorigenicity in both FVB and C57BL/6J backgrounds (data not shown).

Complementary to a previous study [14], we also observed dysregulation of β-catenin signaling in tumor cells, but our finding is significantly different from Gat U et al.'s report. Specifically, we found that both total β-catenin and phosphorylated β-catenin are down-regulated in the tumor, especially in the hair follicle derived cells. In contrast, even though no obvious change was found in majority of tumor cells, active/stable beta-catenin was dramatically up-regulated in dermal papillae. Both studies suggest that dysregulation of β-catenin can lead to hair follicle tumorigenesis, either directly or indirectly. However another study has found that noggin can promote skin tumorigenesis via stimulation of the Wnt and Shh signaling pathways [36], so the precise molecular mechansims underlying tumor formation in our animals remain unclear. However the observation that Nse-Noggin develops a phenotype that closely resembles human trichofolliculomas, not other similar human skin disorders, suggests that disrupted BMP signaling in hair progenitors may specifically contribute to human trichofolliculomas.

## Conclusions

Our observations indicate that inhibition of BMP signaling in P-cadherin positive progenitor cells leads to up-regulation of P-cadherin and down-regulation of E-cadherin with an associated inhibition of hair progenitor cell differentiation. Factors that affect cell migration, such as CD44, are also dynamically regulated which may enable newly generated cells to migrate to form tumors. Understanding the precise roles of BMP signaling in each cell type in the skin will hopefully lead to the development of new therapeutic approaches for using agonist or antagonists of BMP signaling in the treatment of skin and hair growth disorders, such as trichofolliculoma.

## Competing interests

The authors declare that they have no competing interests.

## Authors' contributions

LK: conception and design, collection and assembly of data, data analysis and interpretation, manuscript writing, final approval of manuscript; YL and TM: collection and/or assembly of data; MMB provision of study material, technical advice, JAK: data analysis, manuscript editing, final approval of manuscript. All authors read and approved the final manuscript.
